# Daily Exposure to a Cranberry Polyphenol Oral Rinse Alters the Oral Microbiome but Not Taste Perception in PROP Taster Status Classified Individuals

**DOI:** 10.3390/nu14071492

**Published:** 2022-04-02

**Authors:** Neeta Y. Yousaf, Guojun Wu, Melania Melis, Mariano Mastinu, Cristina Contini, Tiziana Cabras, Iole Tomassini Barbarossa, Liping Zhao, Yan Y. Lam, Beverly J. Tepper

**Affiliations:** 1 Department of Food Science & Center for Sensory Sciences & Innovation, Rutgers University, New Brunswick, NJ 08901, USA; neeta.yousaf@rutgers.edu; 2 Department of Biochemistry and Microbiology, Center for Microbiome, Nutrition, and Health, New Jersey Institute for Food, Nutrition, and Health, Rutgers University, New Brunswick, NJ 08901, USA; gary.guojun.wu@rutgers.edu (G.W.); liping.zhao@rutgers.edu (L.Z.); 3 Department of Biomedical Sciences, University of Cagliari, 09042 Monserrato, Italy; melaniamelis@unica.it (M.M.); mariano.mastinu@unica.it (M.M.); tomassin@unica.it (I.T.B.); 4 Department of Life and Environmental Sciences, University of Cagliari, 09042 Monserrato, Italy; c.contini@unica.it (C.C.); tcabras@unica.it (T.C.); 5 Gut Microbiota and Metabolism Group, Centre for Chinese Herbal Medicine Drug Development, School of Chinese Medicine, Hong Kong Baptist University, Hong Kong, China

**Keywords:** cranberry polyphenols, oral microbiota, salivary proteins, 6-n-propylthiouracil, TAS2R38, PROP phenotype

## Abstract

Diet and salivary proteins influence the composition of the oral microbiome, and recent data suggest that TAS2R38 bitter taste genetics may also play a role. We investigated the effects of daily exposure to a cranberry polyphenol oral rinse on taste perception, salivary proteins, and oral microbiota. 6-n-Propylthiouracil (PROP) super-tasters (ST, *n* = 10) and non-tasters (NT, *n* = 10) rinsed with 30 mL of 0.75 g/L cranberry polyphenol extract (CPE) in spring water, twice daily for 11 days while consuming their habitual diets. The 16S rRNA gene sequencing showed that the NT oral microbiome composition was different than that of STs at baseline (*p* = 0.012) but not after the intervention (*p* = 0.525). Principal coordinates analysis using unweighted UniFrac distance showed that CPE modified microbiome composition in NTs (*p* = 0.023) but not in STs (*p* = 0.096). The intervention also altered specific salivary protein levels (α-amylase, MUC-5B, and selected S-type Cystatins) with no changes in sensory perception. Correlation networks between oral microbiota, salivary proteins, and sensory ratings showed that the ST microbiome had a more complex relationship with salivary proteins, particularly proline-rich proteins, than that in NTs. These findings show that CPE modulated the oral microbiome of NTs to be similar to that of STs, which could have implications for oral health.

## 1. Introduction

Diverse microorganisms inhabit the oral cavity and oral microbiota forms the second-largest microbial community in humans, after the gut [1]. The interactions between oral microbiota, host, and environmental factors influence microbial homeostasis and ultimately human health [2]. The study of taste perception and its relationship with the oral microbiome is an emerging area of research, which has thus far not included an analysis of salivary proteins as an important covariate.

Salivary proteins play an essential role in maintaining a healthy oral environment. Notably, salivary proteins such as basic proline-rich proteins (bPRPs), acidic proline-rich proteins (aPRPs), histatins, cystatins, mucins, statherins, and amylase exhibit antimicrobial and/or antiviral properties and maintain oral lubricity and mineral composition of dental surfaces that collectively form a key cornerstone of oral immunity [3]. Many of these proteins directly or indirectly inhibit bacterial growth via disruption of their enzymes or manipulate bacterial adhesion mechanisms, therefore, affecting biofilm formation [4,5]. In addition to oral immunity, many salivary proteins also influence the taste and mouthfeel perception of polyphenol-rich foods. Salivary proteins bind dietary polyphenols to form complexes that may be partially responsible for the sensation of astringency. In addition, this binding can elicit a cascade of protective mechanisms that defend the oral cavity against physical or chemical damage and may regulate the number of polyphenols available downstream for digestion [6,7]. This is important, as over-consumption of polyphenol-rich foods can be aversive to animals and humans [8]. Nevertheless, feeding a polyphenol-rich diet to mice upregulates key salivary proteins, while reducing aversions to such foods [9]. In a short-term intervention with human subjects, Crawford et al. [10] showed that daily consumption of a polyphenol-rich chocolate-flavored cow’s milk, rich in polyphenols, increased levels of salivary PRPs and Cystatins, and these effects were related to minor changes in taste perception [10]. Therefore, evidence suggests that, in addition to their protective role in the oral cavity, salivary proteins may influence taste perception as well.

Genetic variability in taste sensitivity to the bitterness of 6-n-propylthiouracil (PROP) has been used as an oral marker for the perception and acceptance of bitter and strong-tasting foods with nutritional implications [11,12]. This genetic variability is regulated by polymorphisms in the *TAS2R38* gene, which codes for the bitter taste receptor TAS2R38 [13]. Single-nucleotide polymorphisms in this gene result in structural variation in the TAS2R38 taste receptor, which affects the binding affinity of PROP [14]. Individuals who are homozygous dominant for this gene, phenotypic PROP super-tasters (STs), have a strong binding affinity for PROP and experience intense bitterness from it, while homozygous recessive individuals are non-tasters (NTs), do not bind PROP very well and experience little to no bitterness from it; heterozygous individuals experience intermediate bitterness and are called medium-tasters (MTs) [13,15].

Perception and liking of astringent, polyphenol-rich fruits have been studied in relation to PROP taste responsiveness [16,17,18]. Using cranberry-derived stimuli, Melis and colleagues [19] showed that the saliva of PROP STs had higher levels of acidic PRPs and Cystatins in comparison to that of NTs after tasting unsweetened cranberry juice. In the same study, male NTs reported lower bitterness and astringency from cranberry juice cocktail supplemented with tannic acid (to modify astringency) and gave it higher liking ratings in comparison to PROP STs [19]. More recently, we showed that PROP STs had higher levels of salivary α-amylase in comparison to NTs following oral exposure to cranberry-derived stimuli [20]. In the same study, we also found that male STs had higher levels of bPRPs after oral stimulation when compared to female STs. This pattern was not observed among NTs. These data suggest that the *TAS2R38* phenotype may play a critical role in salivary protein release to cranberry polyphenols. Considering cranberries are abundant in type-A proanthocyanidins (PACs), which are known to have strong oral antimicrobial properties [21,22,23,24,25], these findings have potential implications for immunity and oral health.

Numerous studies in the dental literature have shown that NT adults and children have more dental caries than taster individuals [26,27,28,29]. Although a range of genetic and environmental factors are involved in caries development (diet, oral hygiene, other genes) [30], these observations suggest there may be innate differences in caries risk between NTs and STs. In light of our findings outlined above, showing that oral exposure to cranberry polyphenols elicited higher protein responses in STs compared to NTs, we propose that STs may be better protected against dental caries by virtue of their greater ability to mount a protein response. There is strong evidence that the TAS2R38 receptor plays a sentinel role in protection from upper airway disease and the NT phenotype is associated with greater risk and severity of disease [31,32,33]. Thus, our suggestion that TAS2R38 may serve a role in supporting oral health including maintaining healthy microflora is well supported by parallel studies in upper airway health. Only two previous studies have reported associations between *TAS2R38* genotypes and oral microbiota [34,35], but the focus of these experiments was on taste rather than oral health. Thus, any associations between the PROP taster phenotype in conjunction with the measurement of salivary proteins and oral health remain unexplored.

The present pilot study had three research objectives: (1) to determine if there is variation in the oral microbiome with respect to the PROP phenotype at baseline, (2) if daily rinsing with cranberry-polyphenol extract (CPE) oral rinse would alter the oral microbiome and (3) if the CPE intervention would alter salivary protein profiles or oral sensory perceptions. We hypothesized that the oral microbiome structure would vary with PROP taster phenotype, that the CPE oral rinse would alter the composition and diversity of the oral microbiome, and that these changes would affect salivary proteins and taste perception. Whether these potential changes in the oral microbiome, salivary proteins, and/or taste perception would vary with the PROP phenotype was an additional exploratory aim of this study.

## 2. Materials and Methods

### 2.1. Subject Recruitment

Healthy adults (*n* = 20), between 18–45 years of age were recruited from the Rutgers University community through an email distribution list. Subjects were screened for PROP taste responsiveness; only PROP NTs and STs were admitted into the study; MTs were excluded. Subjects were also screened for general suitability (e.g., demographics, health information) and good oral health status and behaviors, such as brushing teeth at least twice a day and dental cleaning within the last six months. Exclusion criteria included major metabolic diseases (diabetes, kidney disease, etc.), pregnancy, lactation, food allergies, and use of medications that interfere with taste or smell functions (e.g., steroids, antihistamines, or anti-depressants). Subjects were also excluded if they did not have a routine oral health exam within the last six months, if they reported oral pain or discomfort or dental/oral disease (such as periodontitis, infections, recent root canals, etc.), or presence of oral piercings.

The study was approved by the Rutgers University Arts and Sciences Institutional Review Board (Approval#13-309M) and registered in the Clinical Trials database (ClinicalTrials.gov identifier: NCT04107688). All subjects provided written informed consent and were compensated monetarily for their participation.

### 2.2. PROP Taster Status

As part of the screening criteria, participants were classified according to PROP taster status using the paper disk method, which has been previously tested for validity and reliability [19,36,37,38,39]. In brief, in this method, subjects place a filter paper disk impregnated with 1.0 mol/L NaCl (Sodium Chloride, S671-500, Fisher Scientific, Waltham, MA, USA) on the tip of the tongue for 30 s. They rate the taste intensity of the disk using the labeled magnitude scale (LMS), a 100-mm scale anchored with the phrases “barely detectable” to “strongest imaginable”. After a forced 5 min break, this procedure is then repeated with a second paper disk impregnated with 50 mmol/L PROP (6-n-propyl–2-thiouracil, P3755, Sigma-Aldrich, St Louis, MO, USA). Subjects rinse with room-temperature spring water before and in between tasting each paper disk. Subjects are classified NTs if they rate the PROP disk <15 mm on the LMS; they are categorized as STs if they rate the PROP disk >67 on the LMS. All others are classified as MTs. Since NaCl ratings do not vary with PROP taster status [40], these ratings are used as a reference standard to determine the taster status of subjects who give borderline ratings to PROP. This strategy is based on the rationale that NTs give higher ratings to NaCl than to PROP, MTs give equivalent ratings to both stimuli and STs give higher ratings to PROP than they do to NaCl.

### 2.3. Stimuli Preparation

#### 2.3.1. Oral Rinse Samples

An oral rinse solution was formulated using a carrier-free powdered cranberry polyphenol extract, CPE (Ocean Spray Inc., Lakeville-Middleboro, MA, USA) added to spring water at a concentration of 0.75 *w/v* g/L. The control solution was spring water without any added CPE. These samples were dispensed in 20 mL aliquots in sealed amber glass vials. Samples were given to subjects in an opaque aluminum-lined bag to minimize any potential negative effect of heat and light during transport. Subjects were instructed to keep the samples refrigerated and to bring each glass vial to room temperature before use.

#### 2.3.2. Taste Samples

Briefly, cranberry juice (CJ) was made from fresh cranberries (donation, Ocean Spray Inc., Chatsworth, NJ, USA) frozen at −20 °C until use. CJ was made in small batches using a standard recipe where 300 g of berries were defrosted, washed, and cooked on a stovetop under medium heat for 10 min with 648 mL of spring water. The mixture was filtered through cheesecloth and cooled to room temperature and 8.75% *w/v* sugar was added to it to make cranberry juice cocktails (CJC).

CJC with various levels of astringency, as modulated by the addition of cranberry polyphenol extract (CPE), was used as a taste stimulus. Three samples for sensory evaluation were used in this study: CJC (no added CPE), Low CPE (0.50 g/L CPE in CJC), and High CPE (0.80 g/L CPE in CJC). These concentrations were selected after extensive benchtop screening to select a range that was consistent with broad consumer acceptance.

For the Low CPE and High CPE samples, CPE powder in the respective concentrations was mixed directly into freshly prepared CJC. These samples were prepared as needed, the day before subject testing, and refrigerated at 4 °C until 30 min before use. Samples were served at room temperature.

### 2.4. Experimental Procedures

#### 2.4.1. Overall Study Design

**Figure 1:**
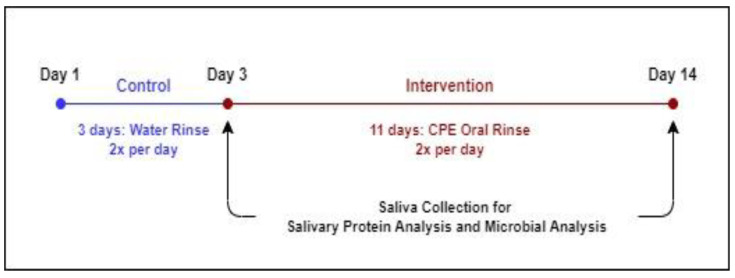
Experimental Timeline.

Subjects participated in a 2-week clinical trial (Figure 1), with a 3-day washout period followed by 11 days of intervention. From Day 1 to Day 3, subjects rinsed their mouths with the spring water control rinse twice a day for 30 s (morning and evening after brushing their teeth). From Days 4 to 14, subjects used the CPE oral rinse, twice a day, following the same procedure as the control. On Days 3 and 14, subjects participated in a test session, in which they provided saliva for oral microbiome profiling as well as proteomic analysis immediately upon arrival at the test site. After saliva collection, subjects were given a short break (10 min) and then asked to evaluate cranberry beverage samples for key sensory attributes. Data resulting from Day 3 is considered the pre-intervention or baseline data, while Day 14 data is referred to as the ‘post-intervention’ data.

During this study, subjects were prohibited from using any other oral rinse products. However, they were not required to change their diet or routines in any way except on saliva collection days. Subjects were instructed to not eat or drink anything except water, smoke, or chew gum on the morning of the test sessions. Test sessions were always conducted between 9:00 and 10:30 a.m. for all subjects to minimize intra-day variation in saliva.

The testing sequence took two weeks to complete.

#### 2.4.2. Saliva Collection and Treatment

At the beginning of the test session, subjects were given a cup to spit into directly. One milliliter of the saliva collected was immediately transferred to a 1.5 mL Eppendorf tube, which was labeled and immediately frozen at −80 °C for oral microbiome profiling. The remaining saliva sample was transferred into two microcentrifuge tubes in an ice bath (0.5 mL per tube). One tube was treated with an acidic solution (0.2% Trifluoroacetic acid, TFA) in a 1:1 *v/v* ratio. These samples were centrifuged at 8000× *g* at 4 °C for 15 min. The supernatant was separated from the pellet and stored at −80 °C until analysis by HPLC-ESI-low resolution-IT-MS. A protease inhibitor cocktail solution (mix of 1 tablet/1.4 mL of cOmplete Protease Inhibitor Cocktail and ammonium bicarbonate 175 mM) (cOmplete^®^, Roche Diagnostics, Branchburg, NJ, USA) was added to the second tube in a 1:2 *v/v* ratio. These samples were stored at −80 °C until analysis by Dot-blot immunoblot procedure for α-amylase and mucins quantification

#### 2.4.3. Oral Microbiome Analysis

A total of 40 saliva samples were collected from 20 subjects (two time points each i.e., pre- and post-intervention). Insufficient genomic DNA was extracted from one saliva sample which was then excluded from subsequent processing and analysis. Approximately 6.55 million usable 16S rRNA V4 amplicon reads were generated (average reads/sample = 167,958 ± 18,572). After denoising and abundance-based filtering, 1031 reliable ASVs (amplicon sequence variants) were retained for further analysis.

Specifically, genomic DNA was extracted from 1 mL aliquots of saliva using protocol Q [41] with minor modifications. Hypervariable region V4 of the 16S rRNA gene was amplified using the 515F and 806R primers modified by Parada et al. [42] and Apprill et al. [43] respectively and sequenced using the Ion GeneStudio S5 (ThermoFisher Scientific, Waltham, MA, USA). Primers were trimmed from the raw reads using cutadapt [44] via QIIME 2 [45]. ASVs [46] were obtained by denoising using the dada2 denoise-single command in QIIME 2 with parameters—p-trim-left 0—p-trunc-len 215. Spurious ASVs were further removed by abundance filtering [47]. A phylogenetic tree of ASVs was built using the QIIME 2 commands alignment mafft, alignment mask, phylogeny fastree, and phylogeny midpoint-root. The taxonomy assignment was performed by the q2-feature-classifier plugin [48] in QIIME 2 based on the silva database (release 132) [49]. The data were then rarified to 76,000 reads/sample for subsequent analyses.

#### 2.4.4. Salivary Protein Analyses

##### Total Protein Content Quantification

Bicinchoninic Acid (BCA) Protein Assay Kit (Sigma-Aldrich, St. Louis, MO, USA) was used to quantify the total protein content of the saliva samples according to the manufacturer’s instructions. We used the total concentration to normalize protein levels for subsequent dot blot immunoblot analyses.

##### Immunoblot Analysis: α-Amylase

The concentration of α-amylase in the saliva samples was estimated semi-quantitatively using the dot-blot technique according to the exact procedure described and followed in [20].

##### Immunoblot Analysis: Mucins

All samples were spotted onto the wet PVDF membrane in triplicate to quantify both MUC-5B and MUC 7. Specifically, each test sample was spotted in a volume of 2 µL (0.38 µg/µL of total protein content). The membrane was blocked with a blocking agent of 5% of BSA (Bovine serum albumin, Sigma Aldrich, St. Louis, MO, USA) in TBS-T buffer (20 mM Tris-HCl pH7.6, 150 mM NaCl, 0.05% Tween 20) for 1 h at room temperature. Subsequently, the membrane was incubated with primary antibody (dilution 1:100; Mucin 7 (1C10): sc-517138 and Mucin 5B (5B#19-2E): sc-21768—Santa Cruz Biotechnology, Inc, Dallas, TX, USA.) in 0.5% of BSA in TBS-T buffer, overnight at 4 °C. Three washes for 5 min with TBS-T buffer were performed and the membrane was incubated for 1 h with secondary antibody (dilution 1:1000; Rabbit anti-Mouse IgG, Secondary Antibody, HRP ThermoFisher Scientific). After three further washes with TBS-T, one wash with TBS (5 min) the membrane was incubated for 5 min with ECL substrate (Clarity Western ECL Substrate, Bio-Rad Laboratories, Inc., Segrate, Italy) for fluorescence signal development and captured on the Chemidoc MP Imaging System (Bio-Rad, Hercules, CA, USA).

##### HPLC-ESI-IT-MS Analysis

Supplementary Table S1 shows the salivary proteins and peptides analyzed in each of the salivary samples in this study. We followed the HPLC-low resolution-ESI-IT-MS technique according to [50] and the exact procedure followed in [20].

#### 2.4.5. Sensory Sample Evaluation

Intensity ratings for key sensory attributes were collected for three samples: CJC supplemented with 0 g/L CPE, 0.5 g/L CPE (Low), and 0.8 g/L CPE (High). A standard, 15-cm line scale end-anchored with the phrases “very weak” to “very strong” was used. Attributes of interest included sweetness, bitterness, sourness, astringency, thickness, cranberry flavor, and overall flavor. Sensory ratings were collected electronically using RedJade data collection software (Curion Insights, Redwood, CA, USA), where the ballot is presented on a computer screen and subjects make their assessments electronically. All samples were served at room temperature. The order of presentation was randomized and there was a forced 5-min break in between sample evaluations.

### 2.5. Data Analysis

XLSTAT (Addinsoft, NY, USA) and R were used to perform all statistical analyses. Normality testing was conducted using Shapiro Wilk’s test and homogeneity of variances was tested using Levene’s test. Where the data were found to be non-normal, a non-parametric test was used.

#### 2.5.1. Oral Microbiome Data Analysis

Alpha diversity indices (Shannon Index, observed ASVs, Faith’s phylogenetic diversity and evenness) and beta diversity distance metrics (weighted and unweighted UniFrac distances [51]) were used to evaluate the overall oral microbiota structure. Principal coordinates analysis (PCoA) and adjusted principal coordinates analysis (aPCoA) were performed by the R packages “ape” [52] and “aPCoA” [53] respectively. A two-tailed Wilcoxon matched-pairs signed-ranks test was used to compare the effect of intervention within the taster groups (e.g., NTs before CPE oral rinse intervention vs. NTs after CPE oral rinse intervention). A two-tailed Mann-Whitney test was performed to compare differences between the taster groups at each of the time points (e.g., NTs vs. STs before the intervention).

ASVs (amplicon sequence variants) shared by >50% of the samples were considered prevalent and selected for guild-based analysis [54]. Specifically, pairwise correlations among the ASVs were calculated using the method described by Bland and Altman [55]. Based on the correlation values, ASVs were clustered into guilds by a dichotomic and tree-based group identification method [54]. A list of prevalent ASVs clustered into each of the guilds can be found in Supplementary Table S2.

Relationships of guilds in the oral microbiome with sensory ratings (using the CPE High sample only) and salivary proteins were examined using the linear mixed effect model in the MaAsLin2 package [56], with subject as a random effect and adjusted for age, gender, and BMI. The correlations between sensory ratings and salivary proteins and those between different guilds in the oral microbiome were determined as described by Bland and Altman [55]. An adjusted *p*-value (*p* < 0.25) was considered significant for all correlation analyses between guilds, sensory ratings, and salivary proteins.

#### 2.5.2. Immunoblot Data and Salivary Proteins

Samples from four subjects were determined to have non-detectable levels of proteins and were therefore eliminated from the analysis. Friedman’s ANOVAs for each protein, comparing pre- and post-intervention data, were conducted to determine the effect of the intervention on salivary proteins (*n* = 16).

#### 2.5.3. Sensory Ratings

Sensory data were analyzed to determine the effects of the intervention on potential changes in the perception of key flavor attributes (sweetness, sourness, bitterness, astringency, thickness, cranberry flavor, and overall flavor) of the taste stimuli. A repeated measured MANOVA was conducted with treatment (pre- or post-intervention) and CPE concentration (CJC, CPE Low, CPE High) as within-subjects factors while PROP taster status (NT or ST) was used as the between-subjects factor.

## 3. Results

Table 1 shows subject characteristics. The subject pool was all Caucasians (*n* = 20). The mean participant age was 21.6 ± 1.1 years and the mean BMI was 24.4 ± 1.1 kg/m^2^.

### 3.1. Oral Microbiome

#### 3.1.1. Comparison of Oral Microbiome between PROP Taster Groups

We first explored alpha diversity metrics to examine oral microbiome differences within NTs and STs. The two PROP taster groups did not differ in alpha diversity at baseline. However, we observed differences in alpha diversity as an effect of the intervention. Specifically, we observed that the CPE oral rinse altered the richness of the microbiome in PROP taster groups as measured by observed ASVs, Faith’s phylogenetic diversity, and evenness. The number of observed ASVs reflects the unique type of ASVs present in a community. Faith’s phylogenetic diversity (FPD) is a qualitative measure of community richness that considers the phylogenetic distances between ASVs, while evenness reflects the abundance distribution of distinct ASVs.

**Figure 2:**
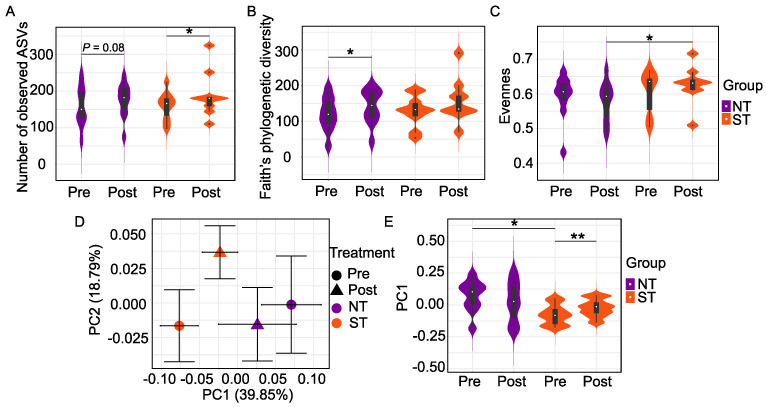
Oral Microbiome differences between PROP taster groups in response to the CPE rinse. Number of observed ASVs (**A**), Faith’s phylogenetic diversity (**B**), and Evenness (**C**) are alpha diversity metrics while beta diversity is based on Weighted UniFrac distance matrices shown in (**D**) as a 2D Principal Coordinate analysis (PCoA) plot and in (**E**) as violin plots. Each point in the PCoA plot represents the mean (±SEM) principal coordinate (PC) score of all individual subjects pre- and post-intervention. In the violin plots, rectangles show the medians and the interquartile ranges (IQRs), the whiskers denote the lowest and highest values that were within 1.5 times the IQR from the first and third quartiles. In NT/ST subjects, Wilcoxon matched-pairs signed-ranks test (two-tailed) was performed to compare pre- and post- intervention samples. Before/After CPE oral rinse, Mann-Whitney test (two-tailed) was performed to compare NT and ST subjects. * *p* < 0.05 and ** *p* < 0.01. Pre-intervention: NT *n* = 10, ST *n* = 10; Post-intervention: NT *n* = 10, ST *n* = 9.

We observed a significantly higher number of ASVs (Figure 2A) in STs (*p* = 0.027) and a similar, but a non-significant trend in NTs (*p* = 0.08, n.s.) at the end of the intervention. In contrast, there was a significant increase in FPD (Figure 2B) in NTs (*p* = 0.049) but a non-significant effect in STs (*p* = 0.164, n.s.) after the intervention. We also observed that the intervention altered the evenness differences (Figure 2C). At baseline, there was no difference between the evenness of the oral microbiome in NTs and STs (*p* = 0.436, n.s.). However, STs had higher evenness in comparison to NTs at the end of the intervention (*p* = 0.035).

Next, we assessed the beta diversity of NTs vs. STs using UniFrac, a phylogenetic method for comparing microbial communities. Unweighted UniFrac distance is a qualitative measure that considers the phylogenetic distances between ASVs but not their abundances. On the other hand, weighted UniFrac distance incorporates the relative abundance of ASVs data and the phylogenetic distances between them and is, therefore, a quantitative metric. We did not find a difference in unweighted UniFrac distance between NTs and STs at baseline or post-intervention in either subject group.

Community dissimilarities based on weighted UniFrac distance were then visualized using a PCoA plot (Figure 2D) and violin plots (Figure 2E). The first two PCs captured 58.64% of the variation in the oral microbiome. PC1, alone, accounted for 39.85% of the variations of the oral microbial community, along which two significant separations between NTs and STs. PERMANOVA test based on weighted UniFrac distance confirmed that NTs and STs had distinct oral microbiota at baseline (*p* = 0.0117) but that the groups did not differ at the end of the intervention (*p* = 0.525, n.s.). Oral microbial community composition among STs at baseline compared to STs post-intervention was significantly different along PC1 (*p* = 0.004, Figure 2E); however, the PERMANOVA analysis of the community composition was not (*p* = 0.373). Similarly, the PERMANOVA test on weighted UniFrac distance in NTs was also non-significant (*p* = 0.678).

#### 3.1.2. Effect of the CPE Rinse Intervention on the Oral Microbiome within PROP Taster Groups

**Figure 3:**
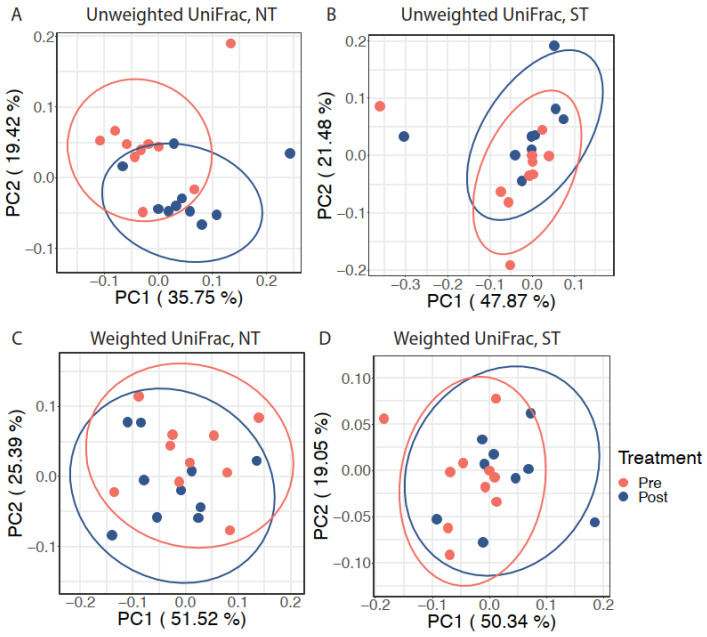
The effect of CPE oral rinse on the oral microbiota with inter-individual variation adjustment. Adjusted principal coordinates analysis (aPCoA) plots on (**A**) unweighted UniFrac distance in NT subjects, (**B**) unweighted UniFrac distance in ST subjects, (**C**) weighted UniFrac distance in NT subjects, and (**D**) weighted UniFrac distance in ST subjects. Ellipses represent 95% confidence intervals.

Next, we explored the effect of the intervention on the oral microbiome within each PROP taster group while accounting for inter-individual variation. After adjusting for subject differences using an adjusted Principal Coordinates Analysis (aPCoA), we observed that the intervention significantly modified the oral microbiota composition in NTs (individual stratified PERMANOVA, *p* = 0.023, Figure 3A) but not in STs (individual stratified PERMANOVA, *p* = 0.096, n.s., Figure 3B) based on unweighted UniFrac distance. However, a similar effect was not observed when considering weighted UniFrac distance (individual stratified PERMANOVA test, *p* = 0.416 for NTs and *p* = 0.168 for STs; Figure 3C and Figure 3D respectively).

#### 3.1.3. Oral microbiome Guilds in Responding to Taster Genotypes and CPE Oral Rinse-Based Analysis of the Oral Microbiome

**Figure 4:**
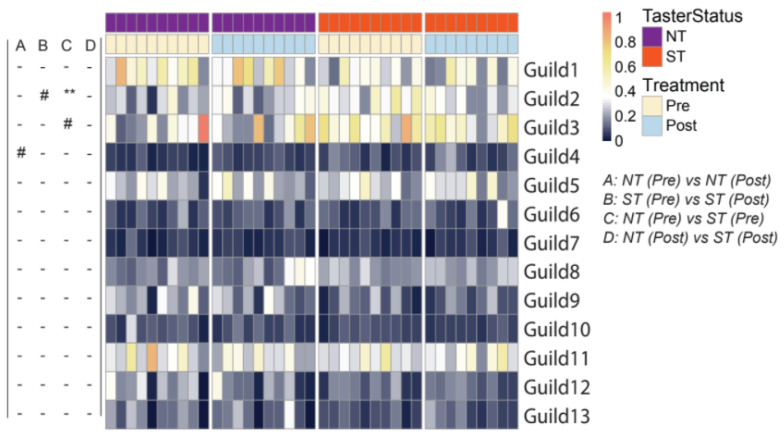
Heatmap showing guild-level differences in the oral microbiome pre- and post- intervention in PROP taster groups. The color of the heatmap represents the abundance of each guild in each sample (arcsine square-root transformed). The left panel shows comparisons between pre- and post-treatment samples in PROP taster groups (A and B) or between NT and ST subjects at the same timepoints (C and D). In NT/ST subjects, Wilcoxon matched-pairs signed-ranks test (two-tailed) was performed to compare pre- and post- treatment samples. Pre-/Post- intervention, Mann-Whitney test (two-tailed) was performed to compare NT and ST subjects. - *p* ≥ 0.1, # *p* < 0.1 and ** *p*< 0.01. ‘-’ indicates no difference between NT and ST subjects. Pre-intervention, NT *n* = 10, ST *n* = 10; Post-intervention, NT *n* = 10, ST *n* = 9.

We further characterized the differences in the oral microbiota associated with PROP taster status and CPE oral rinse by examining ecological interactions among the bacteria. Members of an ecosystem seldom exist in isolation; instead, they develop local interactions and form inter-member organizations to influence the ecosystem’s higher-level patterns and functions [57]. Such inter-member organizations can be considered guilds in which members exhibit co-abundant behavior [54]. We identified the guild structure in 108 prevalent ASVs which were present in more than half of all saliva samples and accounted for ~90% of the total abundance of the oral microbial community. Based on their co-abundance patterns, these 108 ASVs were clustered into 13 guilds (Figure 4; Supplementary Table S2).

Oral microbiota structure at the guild level revealed differences between the PROP taster groups. When compared at baseline, NTs and STs differed in Guild 2 (7 ASVs from *Prevotella*, 2 from *Veillonella*, 1 from *Alloprevotella*, and 1 from *Actinomyces*), whereby STs had a higher abundance of this guild (*p* = 0.005). At the same time point, Guilds 3 and 9 were also found to have a non-significant trend. Guild 3 (3 ASVs from *Prevotella*, 2 from *Veillonella*, 1 from *Atopobium*, and 1 from *Streptococcus*) trended higher in abundance in STs (*p* = 0.063, n.s.), while Guild 9 (2 ASVs from *Porphyromonas*, 1 from *Alloprevotella*, 1 from *Actinomyces graevenitzii*, 1 from *Mannheimia*, 1 from *Capnocytophaga* and 1 from *Actinomycetaceae*) trended lower in abundance in STs in comparison to NTs (*p* = 0.063, n.s.). When comparing guilds post-intervention, the oral microbiome did not differ between taster groups.

When considering the effect of intervention, we observed that Guild 4 (1 ASV from *Eubacterium infirmum*, 1 from *Ruminococcaceae*, 1 from *Leptotrichia*, and 1 from *Lachnoanaerobaculum*) was increased non-significantly in NTs (*p* = 0.084, n.s.), after the intervention. Consistent with the beta diversity analysis, NTs and STs harbored different oral bacterial guilds at baseline. While the intervention affected some guilds, such effects were specific to PROP taster status. Generally, the intervention reduced overall dissimilarities in the oral microbial community between PROP taster groups.

### 3.2. Salivary Proteins

**Figure 5:**
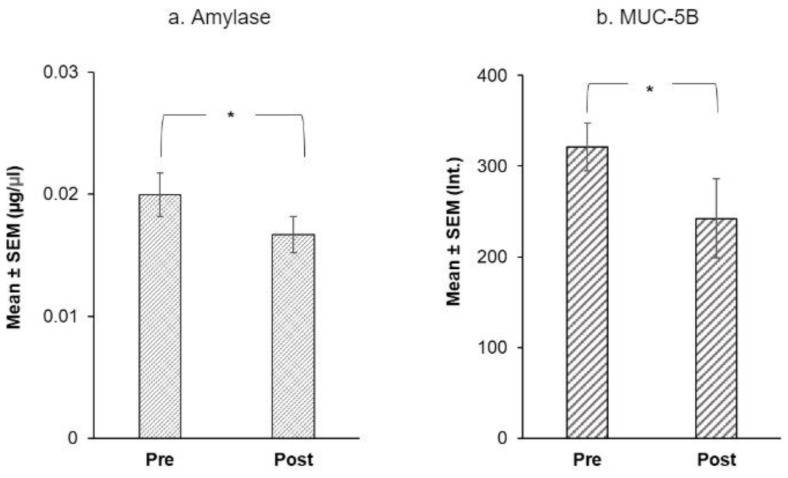
Immunoblot analysis of (**a**) mean α-amylase ± SEM (µg/µL) and (**b**) MUC-5B ± SEM (mean intensity of chemiluminescent signal) before and after the CPE oral rinse intervention. * shows significant effect of treatment (*p* < 0.05) based on Friedman’s ANOVAs (*n* = 20).

**Figure 6:**
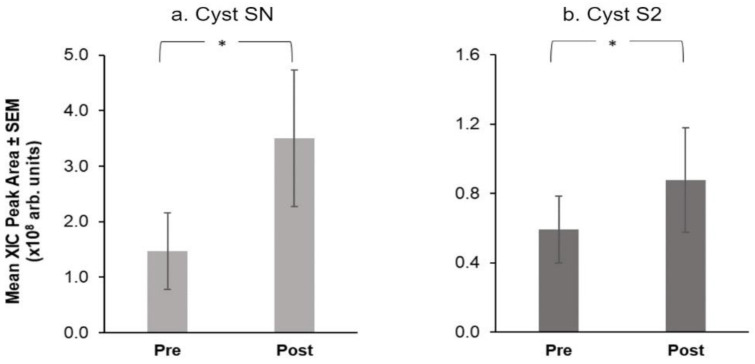
Mean extracted ion current (XIC) peak area ± SEM (×10^8^ arbitrary units) of S-type Cystatins, (**a**) Cyst SN and (**b**) Cyst S2 before and after the CPE oral rinse intervention. * shows significant effect of treatment (*p* < 0.05) based on Friedman’s ANOVAs (*n* = 16).

Using Friedman’s ANOVAs, we evaluated the effect of treatment on salivary proteins. Only four proteins showed a significant difference at the end of the intervention: α-amylase and MUC-5B levels fell after the intervention (Figure 5) while, Cyst SN and Cyst SA levels rose at the end of the intervention (Figure 6).

For α-amylase, mean levels were 0.02 µg/µL at baseline vs. 0.01 µg/µL after the intervention (Q = 5.00, *p* = 0.025) (Figure 5a). Among mucins, MUC-5B (Q = 5.00, *p* = 0.025) was higher at baseline than after the intervention (Figure 5b). There was no effect of treatment on MUC7 levels (*p* = 0.371, n.s.).

Among proteins measured via chromatographic analysis, only two selected S-type Cystatins showed a significant effect of treatment (Figure 6). Specifically, Cyst SN (Q = 5.40, *p* = 0.02) and Cyst S2 (Q = 4.00, *p* = 0.046) showed higher mean levels after the intervention.

There was no significant effect of taster status on differences in salivary proteins.

### 3.3. Sensory Ratings

Sensory ratings of CJC and CJC supplemented with CPE were analyzed at baseline and at the end of the study to examine if the oral rinse intervention altered the sensory perceptions of the subjects. There were no significant differences between taste intensity ratings at the beginning or end of the intervention. There were also no differences observed in ratings with respect to PROP taster status at the beginning vs. the end of the intervention.

**Figure 7:**
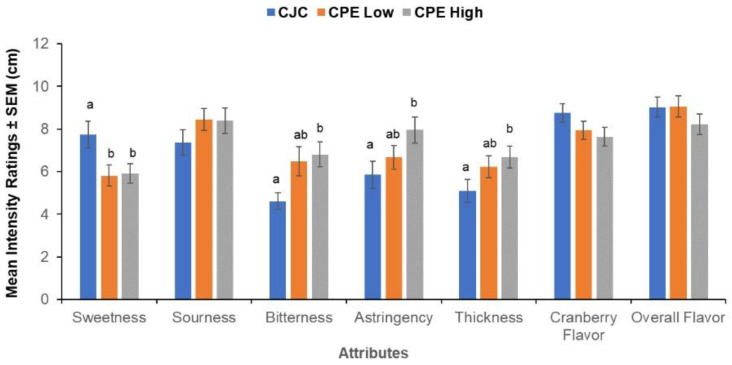
Evaluation of taste samples. Mean intensity ratings ± SEM (cm) for key flavor attributes present in cranberry juice cocktail (CJC) with 0 g/L CPE, 0.5 g/L CPE (Low), and 0.8 g/L CPE (High) of added cranberry polyphenol extract. Letters (a, b) indicate significant differences as a function of concentration (within-subjects) at *p* < 0.05 in a repeated measures MANOVA (*n* = 20).

We found a significant within-subjects effect of CPE concentration (F _(14,62)_ = 2.16, *p* = 0.020) showing that the subjects were able to distinguish the samples with added CPE. This effect arose from the four specific attributes: sweetness, bitterness, astringency, and thickness (Figure 7). CJC with no added CPE received a higher sweetness rating in comparison to the samples supplemented with CPE (F _(2,36)_ = 3.97, *p* = 0.028). In contrast, CPE-supplemented samples were perceived as more bitter (F _(1.43,36)_ = 5.18, *p* = 0.021), astringent (F _(18,36)_ = 4.40, *p* = 0.019) and thicker in comparison to un-supplemented CJC (F _(2,36)_ = 3.67, *p* = 0.035). The samples did not differ significantly in sourness, cranberry flavor, and overall flavor intensities.

### 3.4. Relationship between Oral Microbiome, Salivary Proteins, and Sensory Ratings

**Figure 8:**
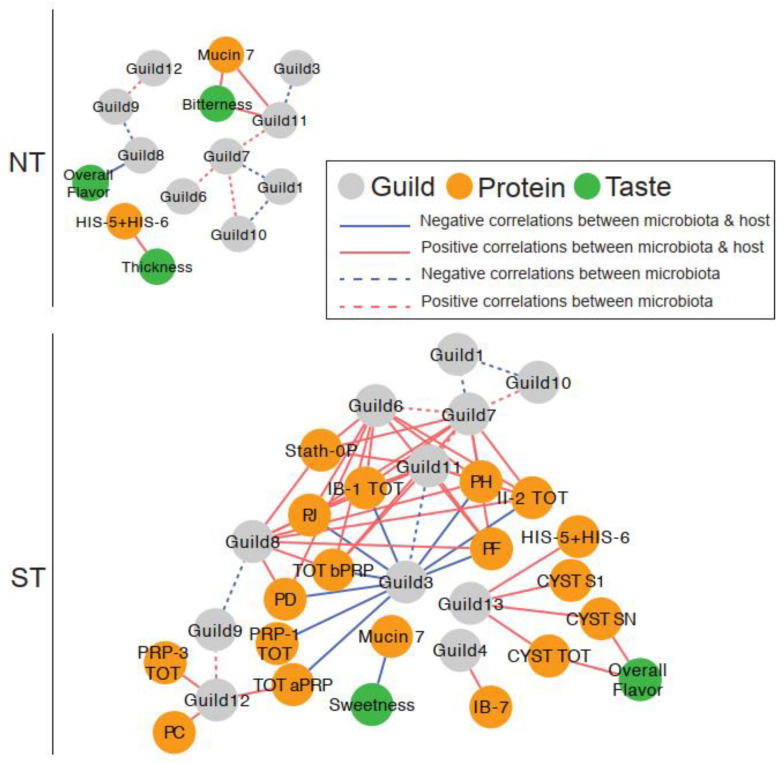
The associations between taste, salivary proteins, and oral microbiota were more complex in PROP STs than in NTs. In the correlation networks, solid edges represent the correlations between taste and guilds, between salivary proteins and guilds, and between taste and salivary proteins. Dashed edges represent the correlations between guilds. The color of the edges represents the positive (red) and negative (blue) correlations. All correlations with adjusted *p* < 0.25 were shown in the networks. Salivary protein abbreviations are explained in Supplementary Table S1.

We constructed correlation networks, one for each PROP taster group, to examine relationships between oral microbiome guilds, salivary proteins, and sensory ratings (Figure 8). Overall, the network for STs was more complex than that for NTs, with more nodes (21 vs. 14 in NTs) and edges (58 vs. 13 in STs).

#### 3.4.1. Microbial Guild- Salivary Protein Associations

The ST network showed extensive interactions between oral bacterial guilds and salivary proteins. In total, 38 positive and nine negative correlations among 11 guilds and 17 salivary proteins were observed. All nine negative correlations were found between Guild 3 and specific proteins from the aPRP and bPRP families. In contrast, Guilds 4, 6, 7, and 8 had several positive correlations with the bPRPs while Guild 12 was positively correlated with aPRPs. In addition, several positive correlations between Guild 13 and selected S-type Cystatins and Histatins were found.

In contrast, there was only one such association in the NT network; Guild 11 was positively correlated with MUC-7. This specific association was not observed in the ST network despite both nodes being present in the network.

#### 3.4.2. Microbial Guild-Sensory Attribute Associations

The oral microbiome also showed interactions with sensory ratings for CJC with the highest CPE concentration, which was only observed in the NT network. Specifically, Guild 11 abundance was positively correlated to bitterness ratings, while Guild 8 was negatively associated with overall flavor.

In the ST network, none of the sensory attributes were correlated with oral bacterial guild abundance.

#### 3.4.3. Salivary Protein-Sensory Attribute Associations

In the NT network, two attributes were correlated with salivary proteins. Bitterness was positively associated with MUC-7, while thickness was negatively associated with Histatin 5–6.

In the ST network, sweetness was negatively correlated with MUC-7, while overall flavor was positively associated with Cyst SN and total S-type Cystatins.

These data show that PROP taster status was associated with distinct interactions between the oral microbiome and salivary proteins, with more complex bacterial guild to salivary protein interactions observed in the STs.

## 4. Discussion

In this study, we found that (1) there was a significant difference in baseline oral microbiome structure between NTs and STs; (2) that CPE oral rinse significantly changed the oral microbiome structure in NTs but not in STs; and (3), the oral microbiome in NTs showed no difference from that in STs after the CPE oral rinse intervention.

The first objective of this study was to determine if there is variation in the oral microbiome with respect to the PROP taster phenotype. Over the last decade, numerous studies have shown that NTs (both adults and children) experience more dental caries and gingival disease than STs [26,27,28]. Subsequent in vitro work suggested that in the presence of oral pathogens, primary gingival epithelial cells derived from the PAV/PAV genotype could mount a stronger immune response, including induction of antimicrobial peptides, than cells from other *TAS2R38* genotypes [58]. This suggests that the oral environment of STs may be better at resisting dysbiosis in the oral microbiome via regulation of innate oral immunity, while that of NTs may be more susceptible to oral disease.

We observed differences in both alpha and beta diversity between NTs and STs at baseline. We clustered co-abundant ASVs into “guilds” based on the principle that a group of ASVs that show consistent co-abundant behavior are likely to contribute to the same ecological function and thus are clustered into one bacterial functional group [54]. We found that the relative abundance of Guild 2, with ASVs in *Prevotella, Veillonella, Alloprevotella,* and *Actinomyces* genera, was significantly higher in STs than in NTs. A further metagenomic analysis is needed to determine the functional significance of the differences observed. Nevertheless, clustering bacteria in guilds based on ASVs as we did here could provide an early insight into oral bacteria most likely to be of functional significance to oral health.

To our knowledge, only two previous studies have investigated the oral microbiome with respect to *TAS2R38*. Sandell and Collado [34] examined the oral microbiome of subjects classified by *TAS2R38* genotypes who resided in Finland or Spain. The purpose of this analysis was to compare the oral microbiome of genotypic groups across the two geographic locations as a proxy for differences in diet and lifestyle. They observed that AVI/AVI and PAV/PAV genotypes (phenotypic PROP NTs and STs, respectively) differed in the oral microbiome at various taxonomic levels across these two geographic locations. In the second study, Cattaneo and colleagues [35] explored variation in the oral microbiome in subjects classified by PROP phenotype (in a single geographic location) and related it to taste sensitivity. Contrary to the findings of the present study, Cattaneo et al. (2019) did not observe differences in alpha or beta diversity of the oral microbiome with respect to PROP taster status but found various genus-level differences.

While both studies [34,35] found differences in the oral microbiome with respect to *TAS2R38* genotype and phenotype, our work differs from these prior works in key aspects. First, there are fundamental differences in how microbiome samples were collected and analyzed in these three oral microbiome studies. The first difference is in the collection sites of the microbiome samples. Sandell and colleagues [34] used buccal swabs while Cattaneo and colleagues [35] used swabs from the tongue dorsum. In contrast, we collected whole-mouth saliva. It is well-known that the microbial composition can vary drastically at different sites in the oral cavity, e.g., microbial composition on the tongue surface can be different than that in saliva [59].

A second major difference lies in the depth of the analysis. Here, we adopted a reference-free analysis of the oral microbiome by using ASVs as basic units of the microbiome instead of operational taxonomic units or reporting genus-level differences. By navigating away from a taxa-based approach, we examined microbiome dissimilarities at a finer resolution by utilizing the dada2 algorithm to differentiate bacterial ASVs. Additionally, we showcase a guild-based analysis, which is based on the ecological framework that bacteria in the oral cavity do not exist in isolation from one another; instead, they may exist in functional communities, which utilize resources in a similar manner and thus proliferate together. Any change in the environment would then affect the entire guild in a similar fashion. Previous work reporting genus-level differences often lumped bacteria together based on taxonomic, but not functional similarity. As a result, important differences may have been missed. Indeed, our guild-based approach demonstrates that even members belonging to the same genus may form co-abundant guilds with other bacteria and exhibit different distributions in NTs and STs. For instance, we observed that ASVs belonging to the same genus (e.g., *Alloprevotella*) were found in both Guilds 2 and 9. However, specific *Alloprevotella* ASVs were more abundant in STs in Guild 2, while other ASVs from the same genus were less abundant in STs in Guild 9. Our approach overcomes various pitfalls of conventional microbiome analysis. We effectively reduced the dimensionality of a large microbiome dataset and also identified the exact ASVs in each guild. Going forward, this strategy will allow for direct comparison between these findings and future work emphasizing the functional ecology of oral microbial communities.

The second objective of this study was to determine if exposure to CPE oral rinse would alter the oral microbiome in NTs and STs. Studies from the oral health literature show that cranberry constituents effectively inhibit bacterial adhesion and co-aggregation in dental plaque and can also reduce the abundance of oral pathogens [25,60]. Specifically, cranberry constituents have been shown to lower counts of the cariogenic pathogen, *Streptococcus mutans*, in a human intervention [24]. Using alpha diversity metrics, we observed some evidence that the CPE oral rinse increased the richness of the oral microbial community in NTs. Our adjusted PCoA model, based on unweighted UniFrac distance, confirmed that changes in microbiome composition were only observed in NTs. It is presently unclear why the CPE oral rinse affected NTs differently than STs. Intriguingly, the oral microbiota of PROP NTs showed no difference from that of STs by the end of the trial. Guild 4 (1 ASV from *Eubacterium infirmum*, 1 from *Ruminococcaceae*, 1 from *Leptotrichia*, and 1 from *Lachnoanaerobaculum*) was increased in NTs. We speculate that the NT microbiome can be characterized as less stable to environmental perturbations such as CPE oral rinse, while the ST microbiome may be more stable to this intervention, conferring beneficial oral health status to STs. The extent to which the stability or resilience of the oral microbiome equates with good oral health status is currently unknown [61,62]. Thus, it is essential to measure markers of host immunity in the future to facilitate a mechanistic understanding of how the CPE induced changes in the microbiome differently for NTs vs. STs. Additionally, this may allow us to relate differences in oral microbiota features to oral health outcomes.

A final objective of the study was to examine potential changes in salivary proteins and taste perception after the intervention. We observed that α-amylase, MUC-5B, and two S-type Cystatins (Cyst SN and Cyst S2) were altered after CPE oral rinse treatment. α-amylase and MUC-5B were lower at the end of the intervention, while the Cystatins increased. Polyphenol ingestion has generally been linked with the up-regulation of several salivary proteins [9,63] in animal models and most recently this has also been demonstrated in humans [10]. Thus, the rise in the two Cystatin levels in our study seems to be consistent with previous findings in the literature. The decreases we observed in levels of α-amylase and MUC-5B were unexpected; the reasons for these outcomes are presently unknown. It is important to note that all previous studies examined daily polyphenol consumption whereas, our work tested daily oral exposure to polyphenols via an oral rinse, not by ingestion. Repeated ingestion of polyphenols may have different physiological effects on some salivary proteins than oral exposure without swallowing. These differences should be examined in future studies.

In terms of taste perception, we found that CJC samples supplemented with CPE were different in sweetness, bitterness, thickness, and astringency compared to un-supplemented CJC. However, we observed no differences between PROP taster groups and no changes in the sensory ratings for any of the CJC samples after the intervention. The latter finding agrees with that of Crawford et al. (2020) who reported only minimal changes in sensory perception after a polyphenol consumption intervention, despite seeing alteration in salivary proteins [10]. It is possible that short-term, oral exposure to polyphenols as used in the present study may not be sufficient to alter taste perception. Based on current evidence, it is uncertain whether polyphenols alter taste perception in humans. This question warrants further attention.

Mechanisms regulating oral health are complex and interlocking. One line of evidence underscores the critical role that taste receptors play in oral health. For example, the TAS2R38 bitter taste receptor serves a protective function by detecting quorum-sensing molecules produced by bacteria and can also regulate oral immunity [32,58]. Having a more functional bitter taste receptor (such as that expressed in PAV/PAV individuals and thus PROP ST individuals) may provide an advantage for oral health, as has been recently shown in animal models with other TAS2R receptors [64]. Another line of evidence reveals that PRPs and Cystatins are involved in oral health and are reported to modulate bacterial attachment to oral mucosa and affect bacterial binding and aggregation [65,66]. We have previously shown that PROP STs have higher salivary levels of specific PRPs after stimulation with astringent stimuli including cranberry-derived polyphenols [19,20]. The exploratory association networks allowed us to combine this disparate information and visualize the interrelationships between the microbial guilds and the salivary proteins in NTs and STs. The networks reveal that interactions between oral microbial guilds and salivary proteins, especially for proline-rich proteins, appear far more complex in the ST oral environment than that in the NT oral environment. These provocative findings are preliminary but could shed light on other relationships e.g., a consequence of differences in these networks could be that food stimuli may affect the oral microbiome differently in NTs in comparison to STs. However, the knowledge of this potential variation is currently extremely limited. This should be investigated in a larger more comprehensive study.

The present study has several limitations. First, this was a short intervention with a small sample size; a longer intervention with a larger sample size may have the potential to capture more profound changes such as gender-related differences. Secondly, we relied on self-reported information to screen subjects for good oral health. Future work should incorporate the DMFT (decayed, missing, filled teeth) index or similar metrics for objective quantification of the oral health status. Third, subjects were instructed to maintain their habitual diets. We did not attempt to control the subjects’ individual diets during the study or assess their dietary behaviors. Fourth, numerous other variables influence taste perception and the oral microbiome, such as salivary flow, pH, and other biomarkers that were not measured in this pilot study. Accounting for these variables in future work, paired with the use of next-generation sequencing would be essential for advancing our understanding of the functional significance of oral microbial communities and their relationship to oral health.

## 5. Conclusions

The present study, to our knowledge, is the first investigation into the interconnectedness of taste, salivary proteins, and the oral microbiome in PROP classified individuals. By adopting the latest tools in bioinformatics, we have demonstrated a way to study these relationships in concert. This presents a path forward for taste researchers interested in studying the highly intertwined nature of taste genetics, dietary behavior, and oral health. Additionally, this study provides important insights for developing interventions for improved oral health without altering taste perception, which should be advantageous from product development and consumer acceptance perspectives.

## Figures and Tables

**Table 1:** Subject Characteristics.

Gender	PROP Classification ^1^	*n*	Age	BMI
			(Years)	(kg/m^2^)
Female (*n* = 12)	NT	7	21.0 ± 1.9	24.7 ± 2.1
	ST	5	20.6 ± 1.3	22.7 ± 1.5
Male (*n* = 8)	NT	3	19.7 ± 1.7	24.8 ± 3.8
	ST	5	24.6 ± 3.4	25.5 ± 2.0

^1^ NT: Non-Tasters, ST: Super-Tasters.

## Data Availability

The raw oral microbiome sequencing data have been deposited to the sequence read archive at NCBI under the BioProject ID PRJNA778221.

## Supplementary Materials

The following supporting information can be downloaded at: https://www.mdpi.com/article/10.3390/nu14071492/s1, Table S1: List of salivary proteins and peptides quantified by HPLC-low resolution-ESI-MS and Table S2: Specific ASVIDs in co-abundant guilds.
