# Supplementary material for: Daily Exposure to a Cranberry Polyphenol Oral Rinse Alters the Oral Microbiome but Not Taste Perception in PROP Taster Status Classified Individuals
Source: Nutrients. 2022 Apr 2;14(7):1492. doi: 10.3390/nu14071492 (PMC9002539; doi:10.3390/nu14071492)
Supplement: Supplementary file 1 [file nutrients-14-01492-s001.zip › nutrients-1636335-supplementary.pdf]

Table S1. List of salivary proteins and peptides quantified by HPLC-low resolution-ESI-MS. Swiss-Prot Code, experimental and theoretical average mass values ± standard deviations (SD) and elution times of the proteins and peptides analyzed.

Protein (Swiss-Prot Code)*	Experimental Average Mass (Da) ± SD (Theoretical)	Elution Time (min ± 0.5)	Abbreviated as:
Acidic proline-rich phosphoproteins family (aPRPs):			TOT aPRP
P-C peptide (P02810)	4370.9 ± 0.4 (4370.8)	13.6-14.5	PC
PRP-1 type di-phosphorylated (P02810)	15515 ± 2 (15514-15515)	22.9-23.3	PRP-1 TOT
PRP-1 type mono-phosphorylated	15435 ± 2 (15434-15435)	23.9-24.3	
PRP-1 type non-phosphorylated	15355 ± 2 (15354-15355)	24.2-24.7	
PRP-1 type tri-phosphorylated	15595 ± 2 (15594-15595)	22.6-22.9	
PRP-3 type di-phosphorylated (P02810)	11161 ± 1 (11161-11162)	23.3-23.8	PRP-3 TOT
PRP-3 type mono-phosphorylated	11081 ± 1 (11081-11082)	23.8-24.2	
PRP-3 type non-phosphorylated	11001 ± 1 (11001-11002)	24.8-25.1	
PRP-3 type di-phosphorylated Des-Arg$_{106}$	11004 ± 1 (11005-11006)	23.5-23.8	
Histatin family (Hists):			TOT HIS
Histatin-1(P015515)	4928.2 ± 0.5 (4928.2)	23.3-23.8	HIS-1
Histatin-1 non-phosphorylated	4848.2 ± 0.5 (4848.2)	23.4-23.8	HIS-1-0P
Histatin-6 (P15516)	3192.4 ± 0.3 (3192.5)	14.0-14.4	HIS-5+HIS-6
Histatin-5 (P15516)	3036.5 ± 0.3 (3036.3)	14.2-14.7	
Basic proline-rich protein family (bPRPs):			TOT bPRP
Ps-1	23460 ± 3 (23459.0)	17.0-18.0	Ps1
P-J	5943.9 ± 0.5 (5943.6)	14.1-14.7	PJ
P-H (P02812/P04280)	5590.2 ± 0.5 (5590.1)	15.0-15.5	PH
P-F (P02812)	5843.0 ± 0.5 (5842.5)	14.3-14.8	PF
P-D (P010163)	6949.5 ± 0.7 (6949.7)	15.2-15.8	PD
II-2 (Tot):			II-2 TOT
- II-2 (P04280)	7609 ± 1 (7609.2)	18.7-19.1	
- II-2 non-phosphorylated	7529 ± 1 (7529.2)	19.5-19.8	
- II-2 Des-Arg$_{75}$	7453 ± 1 (7453.0)	18.8-19.2	
IB-8a (Tot):			IB-8a TOT
- IB-8a (Con1+)	11888 ± 2 (11887.8)	17.1-17.8	
- IB-8a (Con1-)	11898 ± 2 (11896.2)	17.1-17.8	
IB-1 (Tot):			IB-1 TOT
- IB-1 (P04281)	9593 ± 1 (9593.4)	18.8-19.3	
- IB-1 non-phosphorylated	9513 ± 1 (9513.4)	19.4-19.7	
- IB-1 Des-Arg$_{96}$	9437 ± 1 (9437.2)	19.0-19.4	
IB-7 (P02812)	5769.0 ± 0.5 (5769.4)	14.2-14.6	IB-7
Statherin family (Staths)			Stath-TOT
Statherin di-phosphorylated (P02808)	5380.0 ± 0.5 (5379.7)	28.9-29.5	Stath-2P
Statherin mono-phosphorylated	5299.9 ± 0.5 (5299.7)	28.7-29.1	Stath-1P
Statherin non-phosphorylated (P02808)	5220.5 ± 0.5 (5219.7)	28.4-28.8	Stath-0P
Cystatin family (S-type Cysts)			CYST TOT

Cyst S non-phosphorylated (P01036)	14,186 ± 2 (14185)	36.5-37.1	CYST S
Cyst S mono-phosphorylated (S1)	14,266 ± 2 (14265)	36.6-37.1	CYST S1
Cyst S di-phosphorylated (S2)	14,346 ± 2 (14345)	36.8-37.2	CYST S2
Cyst SN (P01037)	14,312 ± 2 (14313)	34.8-35.2	CYST SN
Cyst SA (P09228)	14,347 ± 2 (14346)	38.4-38.9	CYST SA
PB	5972.9 ± 0.5 (5792.7)	29.4-30.5	PB

*Identification was based on the chromatographic behavior and comparison of the experimental mass values with the theoretical ones reported in the Swiss-Prot Data Bank (http://us.expasy.org/tools, accessed on 14 October 2021).

Table S2. Specific ASVIDs in coabundant guilds.

Coabundant Guild (CAG)	ASVID
CAG1	Neisseria ASV032P
CAG1	Prevotella sp. oral taxon 299 str. F0039 ASV0658
CAG1	Prevotella sp. oral taxon 299 str. F0039 ASV065A
CAG1	Rothia ASV00H9
CAG1	Streptococcus ASV004F
CAG1	Actinomyces ASV00CP
CAG1	Lachnoanaerobaculum ASV065V
CAG2	Prevotella melaninogenica ASV0657
CAG2	Prevotella 7 ASV065E
CAG2	Alloprevotella ASV065F
CAG2	Prevotella pallens ASV065I
CAG2	Prevotella pallens ASV065J
CAG2	Prevotella 7 ASV065L
CAG2	Prevotella 6 ASV065M
CAG2	Actinomyces ASV0071
CAG2	Prevotella sp. oral clone P4PB_83 P2 ASV065Y
CAG2	Veillonella ASV066R
CAG2	Veillonella ASV067C
CAG3	Veillonella ASV0065
CAG3	Prevotella 7 ASV065B
CAG3	Veillonella ASV05U4
CAG3	Prevotella 7 ASV065H
CAG3	Prevotella melaninogenica ASV065R
CAG3	Streptococcus ASV007C
CAG3	Atopobium ASV051F
CAG4	[Eubacterium] infirmum ASV065P
CAG4	Ruminococcaceae UCG-014 ASV065W
CAG4	Leptotrichia ASV065X
CAG4	Lachnoanaerobaculum ASV0661
CAG5	Veillonella ASV00BD
CAG5	Selenomonas 3 ASV065G
CAG5	Granulicatella ASV008P
CAG5	Saccharimonadaceae ASV00AI
CAG5	Porphyromonas ASV065Q
CAG5	Aggregatibacter ASV0665
CAG5	Oribacterium sinus ASV066M
CAG5	TM7 phylum sp. oral clone FR058 ASV03VD

CAG5	Selenomonas ASV066U
CAG5	Granulicatella ASV066V
CAG5	Solobacterium ASV066Y
CAG5	Oribacterium ASV0684
CAG6	Rothia ASV02Z9
CAG6	Megasphaera micronuciformis ASV034R
CAG6	Corynebacterium ASV066Q
CAG6	Corynebacterium ASV00AG
CAG6	Selenomonas 3 ASV0680
CAG6	Kingella ASV0682
CAG6	Tannerella ASV068E
CAG6	Prevotella denticola ASV069N
CAG6	Saccharimonadaceae ASV062M
CAG7	Streptococcus ASV0663
CAG7	Fusobacterium ASV066G
CAG7	Mogibacterium ASV067F
CAG7	Fusobacterium ASV068D
CAG7	Selenomonas 3 ASV068Y
CAG7	Paludibacteraceae F0058 ASV06AM
CAG8	Actinomyces ASV006E
CAG8	Campylobacter concisus ASV030Q
CAG8	Alloprevotella ASV065D
CAG8	Actinomyces ASV009P
CAG8	Selenomonas ASV066N
CAG8	Rothia ASV0670
CAG8	Fusobacterium ASV0672
CAG8	Prevotella oris ASV067B
CAG8	Prevotella nigrescens ASV067H
CAG8	Streptococcus ASV068B
CAG8	Prevotella oris ASV068T
CAG8	Streptococcus mutans ASV00DH
CAG8	Scardovia ASV069V
CAG9	Porphyromonas ASV0659
CAG9	Alloprevotella ASV065C
CAG9	Actinomyces graevenitzii F0530 ASV0646
CAG9	Mannheimia ASV05TC
CAG9	Capnocytophaga ASV0677
CAG9	Actinomycetaceae F0332 ASV0687
CAG9	Porphyromonas ASV068V
CAG10	Gemella ASV01LV
CAG10	Actinobacillus porcinus ASV066I
CAG10	Haemophilus ASV066P
CAG10	Prevotella 2 ASV066S
CAG10	Eikenella ASV0671
CAG10	Porphyromonas sp. oral clone HF001 ASV068X
CAG10	(Eubacterium) brachy group ASV06AC
CAG11	Streptococcus ASV00T3
CAG11	Haemophilus ASV00MT
CAG11	Veillonella ASV00QW
CAG11	Rothia ASV0519
CAG11	Actinomyces ASV065U
CAG11	Rothia ASV0337
CAG12	Fusobacterium ASV05VS

CAG12	Bergeyella ASV0668
CAG12	Peptostreptococcus ASV00JI
CAG12	Capnocytophaga ASV066T
CAG12	Clostridiales bacterium oral taxon 075 ASV067D
CAG12	Candidatus Saccharimonas ASV067I
CAG12	Dialister ASV02ZY
CAG12	Peptococcus ASV06AQ
CAG13	Neisseria ASV065K
CAG13	Prevotella 2 ASV065N
CAG13	Lautropia ASV0660
CAG13	Stomatobaculum ASV0666
CAG13	Oribacterium ASV066E
CAG13	Fusobacterium ASV066K
CAG13	Butyrivibrio 2 ASV066W
CAG13	Campylobacter ASV067T
CAG13	Catonella ASV068G
CAG13	Cardiobacterium ASV068S
CAG13	Capnocytophaga gingivalis ASV0692
